# Enhanced Multi-Model Machine Learning-Based Dementia Detection Using a Data Enrichment Framework: Leveraging the Blessing of Dimensionality

**DOI:** 10.3390/bioengineering12060592

**Published:** 2025-05-30

**Authors:** Khomkrit Yongcharoenchaiyasit, Sujitra Arwatchananukul, Georgi Hristov, Punnarumol Temdee

**Affiliations:** 1 Computer and Communication Engineering for Capacity Building Research Center, Chiang Rai 57100, Thailand; 6451501501@lamduan.mfu.ac.th; 2 School of Applied Digital Technology, Mae Fah Luang University, Chiang Rai 57100, Thailand; sujitra.arw@mfu.ac.th; 3 Telecommunications Department, University of Ruse, 7017 Ruse, Bulgaria; ghristov@uni-ruse.bg

**Keywords:** dementia, aortic valve disorder, heart failure, oversampling technique, feature augmentation, blessing of dimensionality

## Abstract

The early diagnosis of dementia, a progressive condition impairing memory, cognition, and functional ability in older adults, is essential for timely intervention and improved patient outcomes. This study proposes a novel multiclass classification that differentiates dementia from other comorbid conditions, specifically cardiovascular diseases, including heart failure and aortic valve disorder, by leveraging the “blessing of dimensionality” to enhance predictive performance while ensuring feature accessibility. Using a dataset of 26,474 electronic health records from two hospitals in Chiang Rai, Thailand, the proposed framework introduced clinically informed feature augmentation to enhance model generalizability. Furthermore, the borderline synthetic minority oversampling technique was employed to address class imbalance, enhancing the model’s performance for minority classes. This study systematically evaluated a suite of machine learning models, including extreme gradient boosting, gradient boosting, random forest, support vector machine, decision trees, k-nearest neighbors, extra trees, and TabNet, across both the original and enriched datasets, with the latter integrating augmented features and synthetic data. Predictive performance was assessed using accuracy, precision, recall, F1 score, area under the receiver operating characteristic curve, and area under the precision–recall curve. The results revealed that all the models exhibited consistent performance improvements with the enriched dataset, affirming the value of dimensionality when guided by domain expertise.

## 1. Introduction

Dementia refers to neurological conditions that progressively impair cognitive functions such as problem-solving, attention, communication, memory, and reasoning [1]. In 2023, it was ranked as the seventh leading cause of death globally, affecting over 55 million people, with nearly 10 million new cases each year [2]. In Thailand, 600,000 older adults were affected in 2015, with projections exceeding 1 million by 2030 and 2 million by 2050 as the population ages [3,4]. Alzheimer’s disease (AD) accounts for 60–70% of dementia cases. Major risk factors include stroke, HIV, hypertension, diabetes, obesity, unhealthy lifestyles, and, most notably, age [2]. Its diagnosis typically relies on medical history, cognitive tests, and blood work, often occurring at a late stage, and there is currently no cure for Alzheimer’s disease [5,6].

Dementia shares several modifiable risk factors with cardiovascular diseases (CVDs), the leading cause of death worldwide, responsible for 32% of all deaths (17.9 million annually). Reduced cerebral blood flow and left atrial dysfunction associated with heart failure and valvular diseases can contribute to cognitive impairment and dementia [7,8]. Heart failure—the primary cause of hospitalization, especially among older adults—presents with symptoms such as shortness of breath, fatigue, and chest pain [9,10,11,12,13]. Declared an emerging epidemic in 1995, its prevalence has continued to rise alongside global aging trends, now affecting an estimated 64.3 million people worldwide [14].

A comprehensive meta-analysis of echocardiographic screening studies found that heart failure prevalence in developed countries reaches 11.8% among individuals aged 65 and older, corresponding to 4.2% in the general population—nearly double the rate reported by registries tracking only diagnosed cases [15]. This discrepancy suggests that over half of heart failure cases may go undetected due to factors such as symptom overlap with conditions like chronic obstructive pulmonary disease, aging, obesity, and limited access to echocardiography in primary care settings [16]. Aortic valve disorder, encompassing regurgitation (backward blood flow) and stenosis (narrowed valve opening), is a common complication of heart failure in individuals aged 50 to 80. While it may develop asymptomatically over 10–20 years, severe cases carry high risks of complications and mortality [17,18,19]. Despite increasing morbidity and mortality rates, complete prevention remains challenging. However, advances in data collection and storage, such as electronic health records (EHRs), present valuable opportunities to address these issues.

Machine learning (ML), increasingly integrated into clinical research, offers robust methods to identify patterns within data through supervised and unsupervised learning approaches [20,21,22,23]. Various ML algorithms have been widely applied in disease prediction, medicinal chemistry, medical imaging, drug interaction analysis, and treatment recommendations [24,25,26,27]. The growing availability of electronic health data has facilitated both novel discoveries and practical implementations that improve patient care [20]. Notably, ML-based disease prediction holds significant promise by detecting unique patterns across health conditions, supporting early diagnosis and treatment. Generally, ML-based dementia prediction typically relies on complex data from advanced diagnostic tools such as magnetic resonance imaging (MRI) [28,29]. However, the high costs and limited accessibility of these tools can hinder widespread application. To address this issue, our previous study [30] and this study utilize biochemical and physiological data from EHRs of older adult patients to promote early dementia detection. To improve generalization and classification performance, this study explores the “blessing of dimensionality” by increasing the dataset’s dimensionality through feature augmentation and data balancing.

In ML, the “blessing of dimensionality” refers to the concept that increasing the number of dimensions can sometimes enhance feature separability. While the “curse of dimensionality” is a common challenge, the blessing highlights scenarios where high-dimensional spaces improve model performance or simplify data structures. To leverage this advantage, additional biochemical and physiological data were collected from another local hospital in Chiang Rai to expand the dataset. Feature augmentation was employed to increase the number of meaningful features, while synthetic data generation was used to address class imbalance by creating more minority class examples.

The primary contribution of this study is to explore the blessing of dimensionality in constructing an effective data enrichment framework for dementia ML-based detection models. To achieve this objective, a diverse and informative dataset was developed through feature augmentation based on medical insights and synthetic data generation to promote transparency and trustworthiness with the details outlined below.

First, comorbidity index mapping techniques were employed to leverage the established correlation between age and the prevalence of comorbid diseases [31]. Diagnostic codes from the International Classification of Diseases, Tenth Revision (ICD-10), in the original dataset were categorized into 17 and 31 distinct comorbidity indices using the Charlson and Elixhauser comorbidity measures, respectively [32,33,34]. Additionally, four mortality risk-scoring algorithms were incorporated, as varying comorbidities associated with each target disease may indicate different mortality risks [35,36,37]. This process generated 52 new features.

Second, rather than relying solely on prediction models to identify feature patterns, prior knowledge-based feature creation techniques were applied to capture relationships between features, particularly those relevant to dementia and CVDs [38]. This approach yielded 16 new features based on medically derived ratios and formulas.

Third, data discretization was conducted to convert 18 demographic and clinical features into interpretable categorical bins, using established medical thresholds. This step aimed to enhance the model’s understanding of the numerical values by providing clinically meaningful categories [39]. Following this process, the dataset contained 108 features—22 baseline features and 86 additional features.

Lastly, to address class imbalance, the borderline synthetic minority oversampling technique (Borderline SMOTE) [40] was utilized. This method was chosen for its proven effectiveness in generating synthetic minority class examples near the decision boundary, leading to a more balanced class distribution while mitigating underfitting.

For this study, the proposed framework was validated on real-world clinical datasets, demonstrating significant improvements in key performance metrics compared to baseline models. Furthermore, the data enrichment framework presented is generalizable, offering potential applicability to a wide range of disease detection tasks and healthcare-related ML applications. The remainder of this paper is organized as follows: Section 2 reviews ML-based models for predicting dementia, heart failure, and aortic valve disorder; Section 3 describes the methodology, including data collection, exclusion criteria, preprocessing, training, hyperparameter tuning, and evaluation metrics; Section 4 presents the model performance comparison; and Section 5 concludes with suggestions for future research.

## 2. Related Studies

This section provides a literature review of relevant studies.

### 2.1. ML-Based Predictive Models with Feature Selection

ML has been widely used in healthcare for its ability to analyze data, uncover patterns, and improve diagnostic systems’ reliability, efficiency, and precision [41]. Feature selection plays a critical role in preventing overfitting and enhancing ML models’ performances. For example, reference [28] demonstrated that a support vector machine (SVM) could effectively predict Alzheimer’s disease using MRI data from 373 samples (150 older adults) obtained from the Open Access Series of Imaging Studies (OASIS). This study used demographic features and brain-related clinical measurements, including the mini-mental state examination (MMSE) score, estimated total intracranial volume (e-TIV), normalized whole brain volume (n-WBV), and atlas scaling factor (ASF). An SVM model with six optimized features achieved 68.75% accuracy and 64.18% precision. Reference [42] applied ML to detect heart failure in a dataset comprising 67,418 healthy controls and 279 patients with undiagnosed heart failure. The dataset included preoperative (medical, surgical, anesthetic) and intraoperative features (monitoring, ventilator use, medications). Extreme gradient boosting (XGBoost) achieved the highest area under the curve (AUC) of 0.873, using 499 preoperative and 263 intraoperative selected features.

A pilot study by reference [43] predicted mortality in 424 heart failure patients (303 survivors, 121 non-survivors) from Fuwai Hospital using ML. The dataset included demographics, vital signs, lab results, echocardiography, comorbidities, and treatments. Feature selection based on correlation, multicollinearity, and clinical relevance yielded 67 features. The XGBoost model achieved 85.4% accuracy, 70.3% precision, 84.3% recall, an F1 score of 76.7%, and an AUC of 0.916, outperforming models using CoxBoost-selected features. Similarly, reference [44] developed an XGBoost-based binary heart disease classification model using a dataset of 303 samples with 76 features from the University of California, Irvine, CA, USA, data repository. Thirteen features—covering demographics, blood tests, fluoroscopy, and electrocardiography—were selected. Combining feature encoding with Bayesian optimization, XGBoost achieved 91.80% accuracy, outperforming RF and ET models. Reference [45] employed XGBoost to predict major cardiovascular and cerebrovascular events, incorporating feature selection, imputation, and normalization techniques. To handle class imbalance, SMOTE and edited nearest neighbors (ENNs) were applied. The model achieved 93.44% accuracy, surpassing RF, KNN, DT, Naive Bayes, and logistic regression models.

For aortic valve disorders, reference [46] developed a model to detect severe aortic stenosis using radiomic features from calcification images of 408 patients (240 severe, 168 non-severe). A total of 128 features were extracted, and feature selection was performed using the least absolute shrinkage and selection operator (LASSO), RF, and XGBoost. The XGBoost model with LASSO-selected features achieved the highest AUC of 0.921, outperforming baseline and other comparative models. For aortic valve regurgitation, reference [47] developed a mortality prediction model for chronic severe cases using data from 1035 older patients. Nineteen features, including demographics, New York Heart Association functional class, comorbidities, baseline symptoms, vital signs, and echocardiography, were selected through a random survival forest (19 features). The conditional survival forest model achieved top AUCs for all-cause mortality (0.857), one-year mortality (0.906), and two-year mortality (0.903), outperforming the ElasticNet Cox model.

### 2.2. ML-Based Predictive Models with Increased Data Dimensionality

While feature reduction is commonly used to prevent overfitting, increasing data dimensionality can also enhance model performance. For dementia prediction, reference [29] expanded their dataset by incorporating additional samples from the OASIS MRI dataset (434 samples from 416 subjects, aged 18–96) and created 21 derived features based on ratios of variables such as age, education, socioeconomic status, MMSE, e-TIV, n-WBV, and ASF. Using the top 20 features, the optimized XGBoost model achieved 85.61% accuracy, 81.40% precision, 77.27% recall, and an F1 score of 79.28%. Similarly, reference [48] developed a model to predict adverse outcomes in chronic heart failure using data from 5004 inpatients in Shanxi, China. The dataset included demographics, medical history, vital signs, treatments, ECG/Echo results, and lab tests. SMOTE and ENNs were applied to balance the dataset and remove noise. The XGBoost model achieved an average AUC of 0.8010 and an F1 score of 0.3673.

In another study, reference [49] built a model to predict chronic heart failure outcomes—mortality, rehospitalization, and major adverse cardiovascular events (MACEs)—using patient-reported outcomes (PROs) from 941 patients across three medical centers. The dataset comprised demographics, clinical data, and responses to a 57-item health questionnaire. SMOTE was used to address class imbalances. Incorporating PROs improved the model’s predictive ability, with XGBoost achieving an AUC of 0.754 for mortality, 0.718 for rehospitalization, and 0.670 for MACEs over a two-year follow-up period. Our previous study [30] introduced a GB multiclass classifier to distinguish dementia from other comorbid conditions such as heart failure and aortic valve stenosis. The model incorporated features from demographics, vital signs, comorbidities, and blood tests, further enhanced with ICD-10 comorbidity indices, in-hospital mortality scores, and cholesterol ratio features. After hyperparameter optimization, the GB model achieved 83.81% accuracy, 82.84% precision, 81.66% recall, an F1 score of 82.09%, and an AUC of 0.9547.

### 2.3. Research Highlights and Proposed Work

A wide range of ML models have been extensively applied in dementia research, addressing both binary and multiclass classification problems. Ensemble learning and deep learning methods have gained prominence in recent years, particularly in dementia classification studies. For example, XGBoost frequently achieves top performance in predictive modeling, and its reliance on specialized features can limit accessibility. To address this, reference [50] developed a model to predict mild cognitive impairment and dementia using survey data from 4975 Korean adults (2018–2020), achieving a median AUC of 0.8185 with XGBoost. In reference [51], the study analyzed data from the Alzheimer’s Disease Neuroimaging Initiative and the National Alzheimer’s Coordinating Center cohorts. It integrated diverse clinical features, including demographics, medical history, neuropsychological tests, MRI, and functional assessments, into a deep learning framework for multiclass dementia classification. The ensemble model, which accepts flexible input combinations, achieved diagnostic performance comparable to expert neurologists, with exceeding 0.9 AUC value. In reference [52], this study demonstrated the potential of deep learning models for automated dementia classification using multimodal data from the DementiaBank’s Pitt Cookie Theft dataset. The baseline models—Wav2vec for audio and Word2vec for text—were evaluated under four data conditions, revealing critical insights into the role of data augmentation. The target task was binary classification of dementia presence. The proposed model achieved strong performances, with AUC scores approaching 0.9, demonstrating the effectiveness of combining audio and linguistic features for early dementia detection. These findings show how important data augmentation is in scenarios with limited and noisy clinical data, where deep learning models typically struggle to generalize due to their high capacity and data dependency.

While deep learning has demonstrated high promise in dementia classification-oriented tasks, its practical application in healthcare settings remains limited due to some constrains. Deep learning algorithms usually require large volumes of well-annotated and high-quality data, which are mostly obtained from advanced laboratory procedures and require comprehensive interpretation. These requirements pose significant challenges for practical applicability, especially for early dementia detection, which this study aims to address. Moreover, it is normal to have class imbalance data from real-world healthcare settings. Therefore, deep learning algorithms still need a data enrichment framework that can provide sufficient variety and balance data to avoid overfitting but ensure model generalization. Therefore, this study mainly concerns ML methods that employ data from typical health care environments where older adults commonly seek care for their existing health conditions. These clinical data are relatively simple to acquire and kept in the EHR. This approach attempts to make it easier for the public to early detection of dementia, especially in the settings in which medical resources are limited. However, challenges such as data dimensionality and class imbalance persist. Especially in comorbid conditions among dementia and CVD (heart failure and aortic valve disorder), it even makes the classification task more challenging due to their overlapping risk factors. The proposed data enrichment framework consists of data augmentation and synthesis generation to provide sufficient effective data for classifications with ML models. Multiple ML models are studied for framework assessment, including a deep learning model for benchmarking.

Therefore, this study aimed to optimize model performance while maintaining accessibility, focusing on the early detection of dementia using biochemical and physiological features commonly recorded in EHRs. More specifically, this study aimed to propose a data enrichment framework for enhancing the performance of multi-models for multiclass classification of dementia, heart failure, and aortic valve disorder, representing new perspectives of dementia detection. To achieve this, this study explored the blessing of dimensionality through the proposed data enrichment framework using feature augmentation and synthetic data generation on original dataset. Medical domain knowledge was applied to generate more meaningful features for feature augmentation, while class imbalance was addressed using the Borderline SMOTE technique, effectively increasing the number of minority class examples. The proposed framework was evaluated with various ML algorithms, including traditional methods (SVM, KNN, DT), ensemble methods (RF, ET, GB, XGBoost), and a deep learning model specialized in tabular data (TabNet). Performance was assessed using precision, recall, F1 score, accuracy, AUC, and area under the precision–recall curve (AUPRC).

## 3. Research Methodology

**Figure 1:**
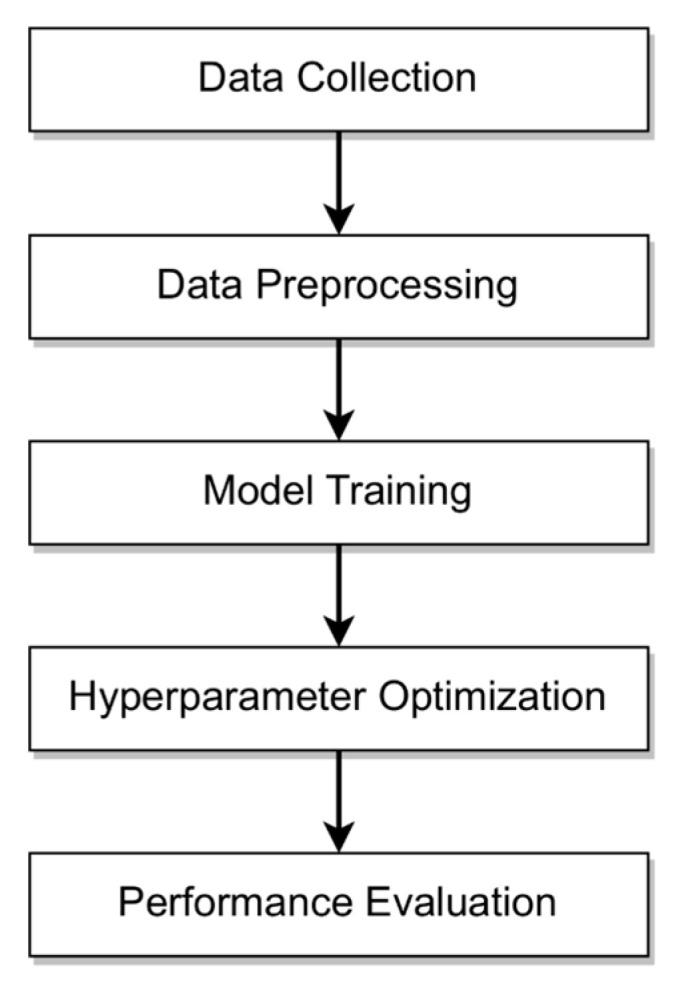
Research methodology including data collection, data preprocessing, model training, hyperparameter optimization, and performance evaluation.

This section outlines the research methodology used to develop the proposed framework, as illustrated in Figure 1.

This section begins by detailing the scope of the data, outlining its characteristics, and defining exclusion criteria to ensure relevance and quality. The following subsection explains the data preprocessing steps in the exact order of execution. Next, the methodology for model training and hyperparameter optimization is described. Finally, the section concludes with a description of the evaluation metrics, ensuring a robust assessment and explainability of the predictions.

### 3.1. Data Collection

This study utilized 26,474 EHRs of older adults diagnosed with dementia (*n* = 7012), heart failure (*n* = 14,249), and aortic valve disorder (*n* = 5213) from two hospitals (Chiangrai Phachanukroh Hospital and Thoeng Hospital) in Chiang Rai province, Thailand. This retrospective study focuses on disease-specific classification. The datasets from two hospitals were combined to enrich the clinical diversity of the target disease. Diagnoses were performed using the first three alphanumeric characters of the International Classification of Diseases, Tenth Revision (ICD-10), codes: F00, F01, F02, and F03 for dementia; I50 for heart failure; and I35 for aortic valve disorder. To ensure data quality and relevance, features that were irrelevant, unusable, or contained complete missingness, often due to human error during data collection, were removed. Nominal features, including gender, smoking status, and alcohol consumption (originally recorded in Thai), were one-hot encoded to enable compatibility with subsequent feature engineering methods and ML algorithms.

The dataset focused on three main categories of features, i.e., demographics, comorbidities, and blood test results, with the latter being the most extensive. Samples were excluded if they met any of the following criteria: missing blood test results (*n* = 9529), age under 60 years (*n* = 3052), or lacking primary diagnosis codes for the target diseases (*n* = 904), as they fell outside the study’s scope. A summary of the dataset characteristics after applying exclusion criteria and outlier handling is provided in Table 1. From the able, a one-way ANOVA test was conducted to assess statistical differences in numerical features, while a chi-square test was performed to evaluate the statistical association between categorical features and the target diseases.

### 3.2. Data Preprocessing

**Figure 2:**
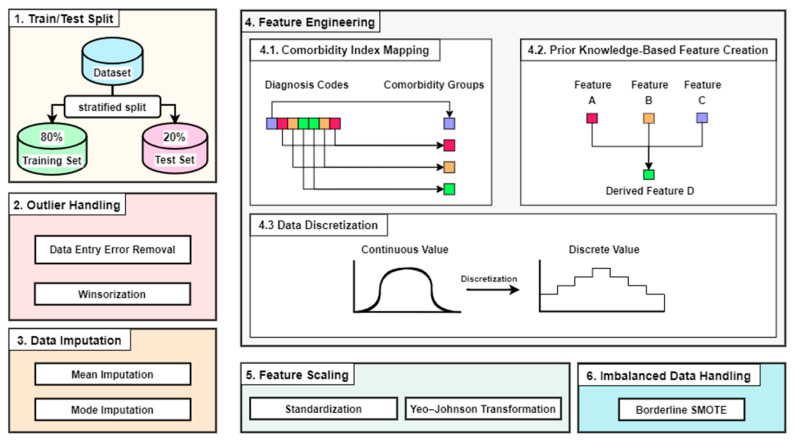
Data preprocessing steps, including outlier handling, data imputation, feature engineering, feature scaling, and imbalanced data handling.

An overview of the data preprocessing steps used in this study is presented in Figure 2.

The details of each data preprocessing step are explained as follows.

#### 3.2.1. Outlier Handling

Based on the observation of the data, two primary steps were taken to limit the influence of outliers that could skew the data distribution, potentially leading to biased interpretations and erroneous analyses. The initial step involved removing feature values that resulted from data entry or measurement errors. This specifically targeted samples with values of zero in numerical features as these values are physiologically improbable. Although the initial step addressed the most apparent outliers, it did not account for all potential outliers. To address the remaining outliers, statistical outlier detection was applied, which involved capping extreme values at the first and ninety ninth percentiles.

#### 3.2.2. Train/Test Split

The dataset was stratified based on the baseline class proportions (25.96% dementia, 59.68% heart failure, and 14.36% aortic valve disorder) to preserve class distribution. It was then randomly split (seed = 42) with 80% for training and validation and 20% of unseen data for testing.

#### 3.2.3. Data Imputation

The dataset contained varying degrees of missing values across numerical and categorical features. Since some ML algorithms cannot handle missing data, imputation was necessary. To address these problems, missing numerical values were imputed using the mean, and missing nominal values were filled with the mode.

#### 3.2.4. Comorbidity Index Mapping

Considering the high prevalence of multiple chronic conditions among older adults [53], diagnostic codes in the dataset were mapped using the ICD-10-based Charlson and Elixhauser comorbidity measures [32,33,34]. These measures classify comorbidities into 17 and 31 categories, respectively. Additionally, four mortality risk-scoring algorithms, which calculate risk based on the weighted sum of present comorbidities, were incorporated [35,36,37]. As a result, 52 comorbidity-related features were added to the dataset.

#### 3.2.5. Prior Knowledge-Based Feature Creation

Incorporating domain-specific knowledge can improve model performance [38,54]. To this end, 16 new features were created, encompassing demographics, vital signs, lipid panels, electrolytes, immune function, kidney function, and cardiovascular disease (CVD) risk. Key features included cholesterol ratios [55,56,57,58], estimated glomerular filtration rate (eGFR) [59,60], neutrophil-to-lymphocyte ratio [61], blood urea nitrogen (BUN)-to-creatinine ratio [62], and CVD risk scores [63]. These additions aimed to enhance the model’s predictive accuracy for CVDs, chronic kidney disease, and metabolic syndromes. The characteristics of the medical domain-guided augmented dataset is shown in Table 2.

#### 3.2.6. Data Discretization

In this study, 18 numerical features, including age, BMI, vital signs, lipid panel results, and fasting blood sugar, were discretized using established thresholds from relevant sources [64,65,66,67,68,69,70,71,72,73,74,75,76,77,78] to improve predictive accuracy.

**Figure 3:**
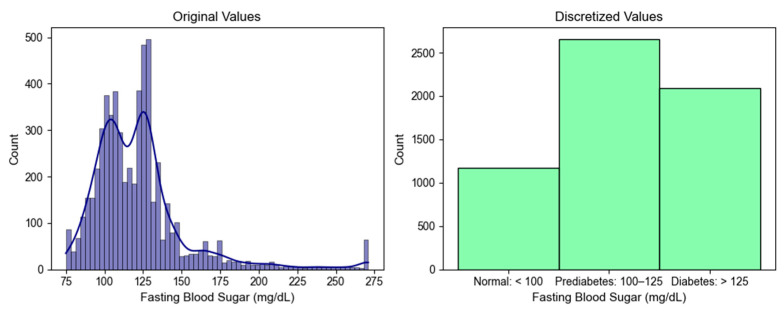
Result of discretization on fasting blood sugar.

Figure 3 illustrates how prior knowledge was applied to discretize fasting blood sugar levels. Guided by prior knowledge, the values were divided into three categories. After this process, the dataset contained a total of 108 features.

#### 3.2.7. Feature Scaling

To address the varying scales of numerical features and ensure equal treatment by the ML algorithms, thereby enhancing model performance, the Yeo–Johnson power transformation method was applied [79]. As shown in Equation (1), this method was selected for its effectiveness in reducing data skewness and transforming features to approximately a normal distribution.

(1) $$\Psi \left(\lambda ,x\right)=\left\{\begin{array}{cc} \frac{{\left(x+1\right)}^{\lambda }-1}{\lambda } & \mathrm{if}\;\lambda \neq 0,x\geq 0\\ \log\left(x+1\right) & \mathrm{if}\;\lambda =0,x\geq 0\\ -\frac{{\left(-x+1\right)}^{2-\lambda }-1}{2-\lambda } & \mathrm{if}\;\lambda \neq 2,x<0\\ -\log\left(-x+1\right) & \mathrm{if}\;\lambda =2,x<0 \end{array}\right.$$

where *x* = the original feature value; *λ* = the power transformation parameter for the selected feature; and Ψ(*λ*;*x*) = the power transformation function of the selected feature for both positive and negative values.

Following this transformation, the data were standardized to have a zero mean and unit variance, as shown in Equation (2).

(2) $$z=\frac{x-\mu }{\sigma }$$

where *x* = the original feature value; *µ* = the mean of the selected feature; *σ* = the standard deviation of the selected feature; and *z* = the standardized value.

#### 3.2.8. Imbalanced Data Handling

To mitigate the bias caused by imbalanced target distributions, Borderline SMOTE [40,80] was applied using seven nearest neighbors and the borderline-1 algorithm. Borderline SMOTE has more potential than traditional SMOTE, K-Means SMOTE, and ADASYN. While traditional SMOTE and K-Means SMOTE generate synthetic samples across the entire minority class space, Borderline SMOTE focuses specifically on samples near decision boundaries, where misclassification typically happens. This targeted approach improves the decision regions between closely related disease classes and helps in enhancing classification performance. Additionally, compared to ADASYN, which adaptively generates samples based on local difficulty but may introduce noise near class overlaps, Borderline SMOTE more effectively preserved class separability, resulting in more robust classification outcomes in the multiclass disease detection task. As a result, the training set expanded from 10,391 to 18,603 samples, with 8212 synthetic samples added, achieving a balanced class distribution of 33.33% per class.

**Figure 4:**
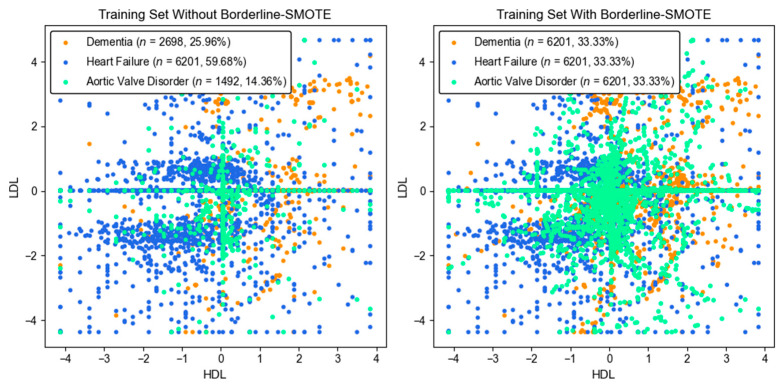
Results of Borderline SMOTE applied to HDL and LDL features of training set, showing the increased number of instances for the dementia and aortic valve disorder classes.

Figure 4 illustrates the effect of Borderline SMOTE on the standardized HDL and LDL, where additional samples for the dementia and aortic valve disorder classes were synthesized to match those of the heart failure class. The left plot shows the original imbalanced dataset, where heart failure dominates, while dementia and aortic valve disorder are underrepresented. In contrast, the right plot demonstrates the effect of Borderline SMOTE, which synthetically balances the dataset by increasing minority class instances so that all three classes have equal representation. This resampling enhances class balances and helps reduce bias in model training.

### 3.3. Model Training and Testing

The multiclass classification models were trained using the Python programming language (version 3.10.8), supported by several key data science libraries, including Scikit-Learn (version 1.2.0), NumPy (version 1.24), Pandas (version 1.5.2), and PyTorch (version 1.13.0). Training was conducted on the resampled dataset to ensure robust model performance without bias from class imbalance. To evaluate the impact of additional features on model performance, each model was trained on two distinct feature sets. The proposed method, XGBoost (version 1.7.2), was compared with a variety of ML models categorized into three groups: traditional classifiers, ensemble learning methods, and deep learning models.

For traditional classifiers, DT mimics human reasoning through a divide-and-conquer strategy, recursively splitting the data to maximize information gain or minimize impurity (using metrics such as information gain or Gini impurity) until stopping conditions, such as node purity or maximum depth, are met [81,82]. The KNN algorithm classifies new data points based on the majority vote of the k closest samples, determined using distance metrics like Manhattan, Euclidean, or Minkowski distances [83]. In contrast, SVM employs kernel functions to map data in a higher-dimensional space, where it identifies the optimal hyperplane that maximizes the margin between classes [84].

For ensemble learning methods, bagging (bootstrap aggregating) enhances model stability and accuracy by training multiple DTs on random subsets of the data, with final predictions determined through majority voting [85]. RF extends this approach by randomly selecting a subset of features at each split, further reducing overfitting and improving generalization [85]. ETs increase randomness by selecting split points at random and utilizing the full dataset, which reduces variance and produces more robust models [86]. Boosting methods iteratively combine weak learners, each correcting the errors of its predecessors, to create a strong ensemble [85]. GB builds an additive approximation of the target function by sequentially fitting models, often DTs, to the residuals of previous iterations [87]. XGBoost improves upon GB by integrating regularization to enhance generalization, using a column-based storage structure for computational efficiency, and selecting candidate splits from data percentiles. These advancements result in faster processing and higher accuracy, particularly with large datasets [87].

Finally, while deep neural networks excel in processing images, text, and audio, they often struggle with tabular data. TabNet [88] addresses this limitation by employing instance-wise feature selection to determine hyperplane decision boundaries. Its sequential multi-step architecture, combined with an attention mechanism, dynamically focuses on semantically meaningful features, thereby enhancing nonlinear learning.

### 3.4. Performance Metrics

This study used a confusion matrix to evaluate model performance, deriving key metrics as shown in Equations (3)–(8). In these equations, TP = true positive (correctly predicted target class); TN = true negative (correctly predicted non-target class); FP = false positive (incorrectly predicted target class); and FN = false negative (incorrectly predicted non-target class).

(3) $$\mathrm{Precision}=\frac{\mathrm{TP}}{\mathrm{TP}+\mathrm{FP}}$$

Precision measures the reliability of positive predictions, making it vital in healthcare applications. High precision ensures accurate diagnoses and reduces the risk of over-diagnosis and unnecessary interventions, while low precision may lead to excessive false alarms.

(4) $$\mathrm{Recall}=\frac{\mathrm{TP}}{\mathrm{TP}+\mathrm{FN}}$$

Recall is essential in healthcare contexts, where minimizing FN is crucial to avoid the severe consequences of misclassification or missed diagnoses.

(5) $$\mathrm{Accuracy}=\frac{\mathrm{TP}+\mathrm{TN}}{\mathrm{TP}+\mathrm{FP}+\mathrm{TN}+\mathrm{FN}}$$

Accuracy reflects a model’s overall correctness by calculating the proportion of true predictions (TP and TN) out of all predictions. In disease prediction, higher accuracy indicates improved diagnostic effectiveness.

(6) $$F1\;\mathrm{Score}=2\times \frac{\mathrm{Precision}\times \mathrm{Recall}}{\mathrm{Precision}+\mathrm{Recall}}$$

The F1 score, the harmonic mean of precision and recall, balances model performance. In healthcare, focusing solely on precision may increase false negatives (missing true cases), while prioritizing recall may lead to more false positives. The F1 score addresses this trade-off by considering both metrics.

(7) $$\mathrm{True}\;\mathrm{Positive}\;\mathrm{Rate}\;(\mathrm{TPR})=\frac{\mathrm{TP}}{\mathrm{TP}+\mathrm{FN}}$$

(8) $$\mathrm{False}\;\mathrm{Positive}\;\mathrm{Rate}\;(\mathrm{FPR})=\frac{\mathrm{FP}}{\mathrm{TN}+\mathrm{FP}}$$

The receiver operating characteristic (ROC) curve plots the TPR against the FPR, with the AUC ranging from 0 to 1. A higher AUC value, closer to 1, indicates better overall model performance. For imbalanced datasets, the precision–recall (PR) curve, which plots precision against recall, provides a more realistic evaluation of model effectiveness. The curve is summarized by the average precision (AP) score, which better reflects the model’s ability to correctly identify positive classes in scenarios where class imbalance is significant.

### 3.5. Hyperparameter Optimization

This study employed the Optuna framework [89] for hyperparameter tuning, incorporating a 10-fold stratified cross-validation strategy. For each model, 100 optimization trials were conducted, with the objective of maximizing the macro-averaged F1 score. The optimization process utilized a tree-structured Parzen estimator within a predefined search space.

## 4. Experimental Results and Discussion

This section presents a comparison of predictive models built using various feature combinations. Each performance metric was evaluated on an unseen test set to assess the models’ generalization capabilities. Variability in performance, represented by the mean and standard deviation, was calculated using stratified 10-fold cross-validation and is reported in Table 3. To validate the model’s superiority, statistical significance and effect sizes were analyzed using the one-tailed Wilcoxon signed-rank test and Cliff’s Delta, respectively. The results from the test set evaluation are provided in Table 4.

### 4.1. Model Comparison Based on Precision

The analysis began with the evaluation of each model’s cross-validated macro-averaged precision (refer to Table 3). The integration of additional features significantly enhanced the performance of XGBoost, GB, TabNet, and SVM (*p*-value < 0.01), with effect sizes of 0.62, 0.74, 1.00, and 0.64, respectively.

For the unseen test data (Table 4), all models performed better with full feature set. In addition, XGBoost achieved the highest macro-averaged precision of 90.20% using the baseline features. After incorporating additional features, the model maintained this positive trend observed during cross-validation, achieving a macro-averaged precision of 91.42%, representing a 1.22% improvement.

### 4.2. Model Comparison Based on Recall

Across different validation folds (Table 3), all models consistently achieved a high macro-averaged recall across both feature sets, significantly outperforming most models (*p*-value < 0.01, large effect size > 0.474). The only exceptions were the RF model with baseline features and the GB model with the full feature set, where no significant differences were observed. Incorporating additional features led to significant improvements in the cross-validated recall for XGBoost, GB, TabNet, and SVM (*p*-value < 0.05). This improvement reflects a reduction in FNs, as indicated by the effect size values of 0.52, 0.68, 0.80, and 0.36, respectively.

For the unseen test set using baseline features (Table 4), all models also performed better. Especially, XGBoost achieved the highest macro-averaged recall of 87.88%. After incorporating additional features, this model improved further, attaining a macro-averaged recall of 89.08%, representing a 1.20% improvement.

### 4.3. Model Comparison Based on F1 Score

Across different validation folds (Table 3), all models consistently achieved a higher macro-averaged F1 score across both feature sets. The integration of additional features notably improved the balance between precision and recall for XGBoost, GB, TabNet, and SVM (*p*-value < 0.05), with large effect size values of 0.60, 0.80, 1.00, and 0.50, respectively.

For the unseen test set using baseline features (Table 4), XGBoost achieved the highest macro-averaged F1 score of 88.97%. After incorporating additional features, this model further improved, attaining a macro-averaged F1 score of 90.19%, thereby reflecting a 1.22% improvement. However, all models performed better with the full feature set.

### 4.4. Model Comparison Based on Accuracy

As shown in Table 3, the inclusion of additional features significantly improved the accuracy of XGBoost, GB, TabNet, and SVM (*p*-value < 0.01), as evidenced by increased TPs and TNs and reduced FPs and FNs. The corresponding effect sizes were 0.66, 0.82, 1.00, and 0.78, respectively.

For the unseen test set (Table 4), all models performed better in terms of accuracy. However, XGBoost produced the most accurate predictions using the baseline feature set, achieving 91.76% accuracy. This model further improved with the full feature set, reaching 92.73% accuracy, representing a 0.97% improvement.

**Figure 5:**
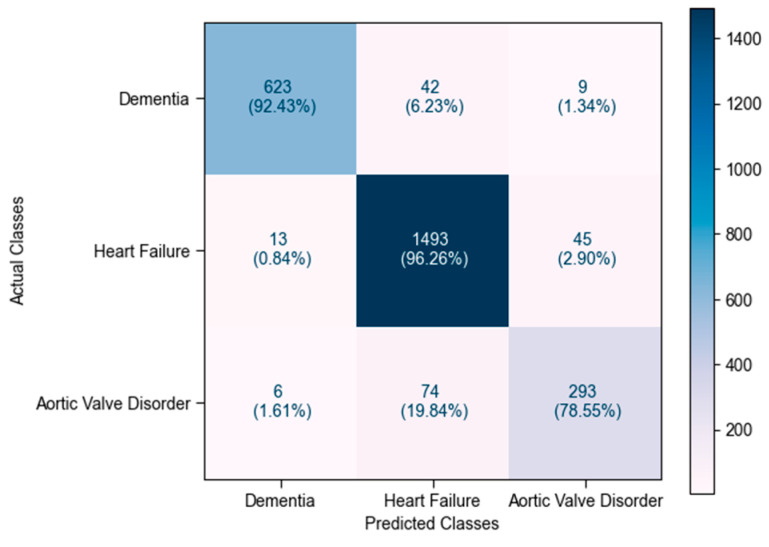
XGBoost-based confusion matrix after using full feature set, indicating best performance among all models.

Considering the XGBoost model in greater detail, as illustrated in Figure 5, it successfully captured 96.26% of heart failure records, 92.43% of dementia records, and 78.55% of aortic valve disorder records. Notably, 19.84% of aortic valve disorder records were misclassified as heart failure, which may be attributed to the clinical similarity between the two conditions, particularly in terms of overlapping feature values.

### 4.5. Model Comparison Based on ROC Curve

In this study, the one-vs-rest technique was applied to transform the multiclass problem into multiple binary classifications, enabling the calculation of the unweighted mean AUC score as a prediction metric. Across different validation folds (Table 3), the integration of additional features significantly improved the discriminative performance of XGBoost, GB, RF, ETs, TabNet, SVM, and KNN. These improvements were statistically significant (*p*-value < 0.01), except for TabNet (*p*-value < 0.05), with large effect size values of 0.96, 0.96, 0.76, 1.00, 0.70, and 0.56, respectively.

Focusing on the unseen test set (Table 4), all models performed better in terms of the ROC curve. Especially, the XGBoost model achieved the highest AUC of 0.9754 using the baseline features. Incorporating additional features further enhanced performance, with the XGBoost model reaching an AUC of 0.9816, representing a 0.0062 improvement.

**Figure 6:**
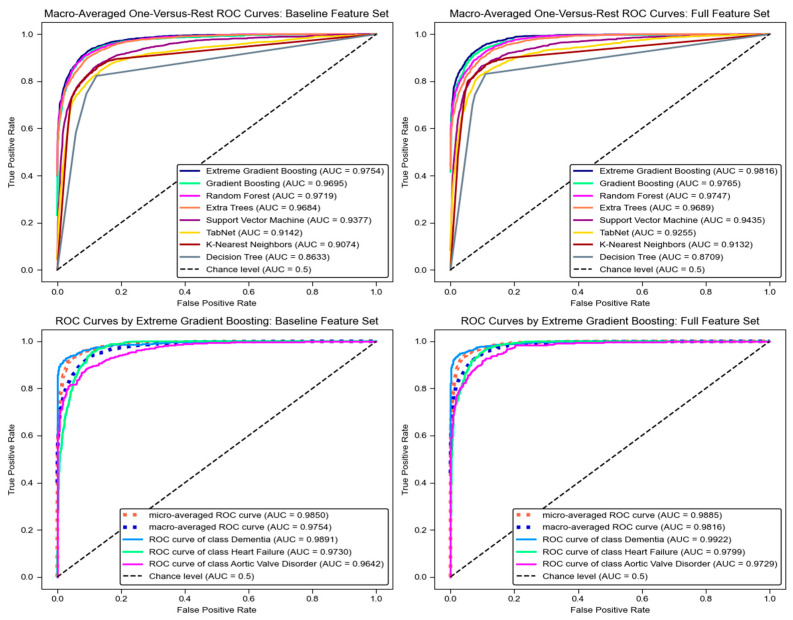
ROC curves demonstrating that XGBoost model achieved superior classification performance compared to other enhanced models.

Furthermore, the ROC curves in Figure 6 demonstrate that the XGBoost model obtained excellent classification performance. Using the full feature set, the dementia class demonstrated the strongest performance, with an AUC of 0.9922, followed closely by heart failure, with an AUC of 0.9799, and the aortic valve disorder class, with an AUC of 0.9729.

### 4.6. Model Comparison Based on PR Curve

Across different validation folds (Table 3), the inclusion of additional features significantly improved the performance of XGBoost, GB, RF, and KNN (*p*-value < 0.01) and SVM (*p*-value < 0.05). The XGBoost achieved the highest AUPRC across both feature sets, outperforming all other models (*p*-value < 0.01, large effect size > 0.474). These improvements indicate better balance between precision and recall across different thresholds, with large effect size values of 0.88, 0.92, 0.60, and 0.64, respectively.

For the unseen test set (Table 4), most models experienced improvements in AUPRC scores with the full feature set. In addition, the XGBoost model achieved the highest AUPRC of 0.9481 using the baseline feature set. Notably, the AUPRC of the XGBoost model further improved to 0.9570, exhibiting an improvement of 0.0089. However, consistent with the F1 score results, which reveal a model’s true potential under class imbalance, the ET model did not show performance improvement with the full feature set, achieving an AUPRC of 0.9341, a decrease of −0.0028.

**Figure 7:**
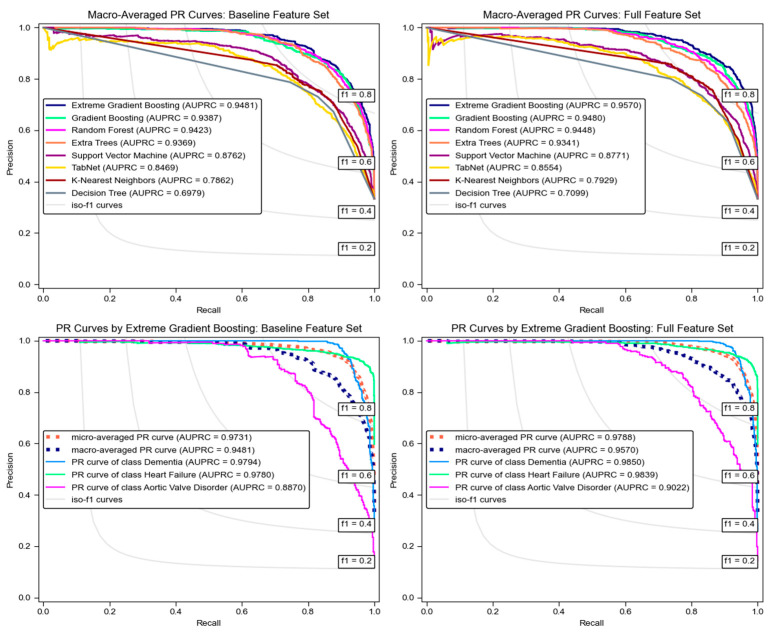
PR curves indicating that XGBoost model demonstrated strong balance between precision and recall compared to other enhanced models.

The XGBoost model demonstrated a strong balance between precision and recall, as illustrated in Figure 7.

As shown in Figure 7, the dementia class achieved the highest AUPRC of 0.9794 using the baseline feature set, which improved to 0.9850 with the full feature set, reflecting a 0.0056 increase. This indicates the model’s excellent ability to distinguish dementia cases. Although the aortic valve disorder class recorded the lowest AUPRC compared to the other classes, its performance remained acceptable, especially after incorporating the full feature set, which improved the AUPRC to 0.9022.

### 4.7. Discussion of Overall Results

The summary of model performance on the test set, as shown in Table 4, indicates that ensemble methods are among the top performers across both feature sets. Their superior classification performance can be attributed to their ability to reduce variance and bias by combining the decisions of multiple models. Specifically, in RF, the use of bootstrap replicas creates diversified subsamples, which helps reduce variance and improve generalization. In contrast, ETs use the entire training set for input sampling, which lowers bias but can increase variance. This trade-off partially explains ET’s slightly lower or stagnant performance compared to RF across most metrics. The selection of cutoff points during the tree-building process is critical for both RF and ET as it directly affects tree quality and the effectiveness of binary splits in the feature space. Poorly chosen split points can degrade classification performance, highlighting the importance of optimal split selection in achieving robust predictions.

Boosting-based models, particularly XGBoost, demonstrated exceptional performance in this study. XGBoost, being an excellent boosting-based model, achieved the best scores, especially when utilizing all available features. Unlike bagging methods, which rely on majority voting from multiple independent trees, XGBoost employs a sequential learning approach where each model is trained to correct the errors of its predecessor. This iterative process uses random data samples and an optimized learning rate, leading to progressively improved predictions and enhanced overall performance. A key contributor to XGBoost’s success was the combination of the “lossguide” growth policy and the “approx” tree method, which proved highly effective in addressing generalization challenges, particularly those caused by uninformative features and underrepresented classes. The “lossguide” policy prioritized splits that maximized reductions in overall loss, making it especially useful when feature engineering and Borderline SMOTE were applied to increase data dimensionality. The “approx” algorithm further enhanced performance by accelerating tree construction, enabling faster convergence without sacrificing accuracy on unseen data. In contrast, GB employs a depth-wise growth policy, which can be less efficient in identifying optimal splits. Unlike the “lossguide” policy, the depth-wise approach does not directly account for the impact of splits on the loss function.

Among the traditional models, SVM demonstrated notable performance, often comparable to TabNet. This performance can be attributed to the nonlinear relationships present in the original features, which initially challenged SVM’s ability to identify an optimal hyperplane for classification. The addition of new features that captured these nonlinear patterns improved data linearity, making it easier for SVM to separate classes and ultimately enhancing its predictive performance.

The TabNet model demonstrated lower performance compared to other models in this study. As a deep learning architecture, TabNet typically requires a larger and more diverse dataset to effectively capture complex patterns. The limited size and diversity of the dataset enriched in this study may have hindered its learning capability. Additionally, TabNet’s attention-based feature selection mechanism may not have been fully compatible with the dataset’s characteristics, potentially leading to suboptimal feature utilization. The training approach, which involved separate training and validation sets, could have further contributed to overfitting, particularly if the validation set was not representative of the overall dataset. Despite these challenges, incorporating the full feature set significantly improved TabNet’s classification performance. For example, the model exhibited a 2.51% improvement in precision and a 2.04% improvement in accuracy, outperforming the relatively minor gains observed in other models. These results highlight TabNet’s promising potential to achieve even better performance with access to larger and more varied datasets.

Future research will focus on applying the proposed framework of dimension expansion and class balancing to a diverse range of disease classifications, including rare and complex conditions, to rigorously evaluate its generalization capabilities. Comparative analyses with state-of-the-art methods, especially the neural network for tabular data, will be conducted to benchmark performance, while also examining the ethical implications and potential biases in artificial intelligence-driven disease classification.

### 4.8. Feature Importance Based on SHAP Values

**Figure 8:**
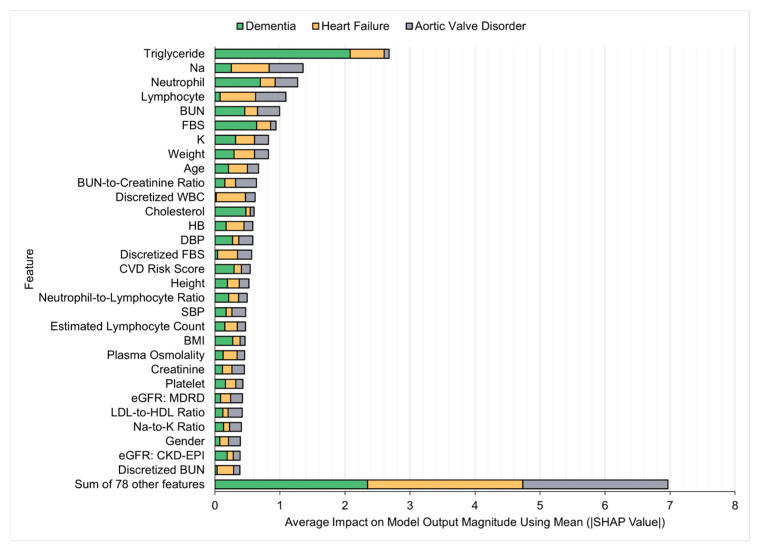
Top 30 features for XGBoost using SHAP values based on the full feature set.

To understand the impact of individual features on the model’s predictions, SHapley Additive exPlanations (SHAP) was applied to the XGBoost model, which performed best with the full feature set. SHAP’s model-agnostic approach provides insights into feature contributions at both local and global levels, leveraging Shapley values from cooperative game theory to offer a comprehensive analysis of the model’s behavior [89]. The top 30 features, ranked by their mean absolute SHAP values, are summarized in Figure 8.

The results demonstrate a consistent influence of certain features across the prediction of various target diseases. From Figure 8, it is evident that triglyceride levels emerged as a significant predictor for all target diseases examined, particularly dementia, highlighting the biomarker’s relevance to cardiovascular and metabolic health. Lymphocyte and neutrophil levels were consistently notable, supporting their established associations with adverse outcomes across various conditions. Several derived features, such as the blood urea nitrogen-to-creatinine ratio, plasma osmolality, LDL-to-HDL ratio, sodium-to-potassium ratio, CVD risk score, and eGFR formulas, provided nuanced insights but had relatively minor overall impact compared to their original features. The discretized white blood cell (WBC) count and fasting blood sugar showed moderate importance in predicting heart failure and aortic valve disorder. This suggests that their discretized forms offered more interpretable and predictive value than their original continuous representations. Importantly, the superior performance achieved with the full feature set was largely attributed to the collective contribution of all features. While many features were individually less significant, their combined effect notably enhanced the model’s predictive capability. The contribution from the remaining 78 features indicates that excluding these seemingly minor predictors could lead to suboptimal results.

## 5. Conclusions

This study presents a novel data enrichment framework for ML-based dementia detection models that leverages the “blessing of dimensionality”. The novelty of this work lies in its novel perspective for distinguishing dementia from its comorbid conditions, including heart failure and aortic valve disorder. The biochemical and physiological dataset was sourced from the EHRs of two hospitals in Chiang Rai, Thailand. By leveraging the blessing of dimensionality, the dataset was enriched through medical knowledge-based feature engineering, which involved comorbidity index mapping, prior knowledge-based feature augmentation, data discretization, and the application of Borderline SMOTE to handle class imbalance. This data enrichment framework resulted in a more balanced dataset and a more meaningful and diverse feature set, playing a crucial role in improving the model’s multiclass classification performance. For the assessment, various supervised learning models, including DT, SVM, KNN, GB, RF, XGBoost, ETs, and TabNet, were evaluated. With additional features and synthetic training examples, all hyperparameter-optimized models demonstrated enhanced predictive performance across all evaluation metrics. Notably, the XGBoost model outperformed the other models, highlighting the value of high-dimensional data in clinical ML applications.

## Figures and Tables

**Table 1:** Baseline dataset characteristics.

Feature	Description	Dementia (*n* = 3372)	Heart Failure (*n* = 7752)	Aortic Valve Disorder (*n* = 1865)	*p*-Value
Age	Age in years	74.83 ± 8.38	73.43 ± 8.61	71.50 ± 7.83	1.88 × 10^−41^
Weight	Body weight in kg	51.86 ± 7.94	58.25 ± 8.56	53.64 ± 8.01	6.82 × 10^−86^
Height	Height in cm	155.29 ± 7.86	154.80 ± 8.59	156.23 ± 8.03	2.57 × 10^−11^
Male (*n*, %)	Biological characteristics of sex	1551 (46.00)	3307 (42.66)	1014 (54.37)	4.79 × 10^−19^
Female (*n*, %)	Biological characteristics of sex	1821 (54.00)	4445 (57.34)	851 (45.63)	4.79 × 10^−19^
SBP	Systolic blood pressure in mmHg	128.63 ± 20.59	127.36 ± 16.46	131.02 ± 14.95	2.85 × 10^−8^
DBP	Diastolic blood pressure in mmHg	70.09 ± 12.36	68.10 ± 10.85	67.39 ± 9.56	3.41 × 10^−6^
FBS	Fasting blood sugar in mg/dL	121.22 ± 24.48	125.80 ± 34.26	106.18 ± 20.08	3.92 × 10^−72^
Cholesterol	Total cholesterol in mg/dL	183.52 ± 34.00	163.64 ± 27.87	163.21 ± 23.69	6.89 × 10^−124^
HDL	High-density lipoprotein (HDL) cholesterol in mg/dL	48.30 ± 8.54	40.81 ± 8.54	44.64 ± 7.66	8.10 × 10^−200^
LDL	Low-density lipoprotein (LDL) cholesterol in mg/dL	120.09 ± 27.40	111.16 ± 23.46	107.59 ± 17.96	4.17 × 10^−35^
Triglyceride	Triglyceride level in mg/dL	116.18 ± 26.59	117.57 ± 39.72	111.41 ± 35.43	2.59 × 10^−6^
BUN	Blood urea nitrogen (BUN) in mg/dL	19.65 ± 8.13	29.65 ± 20.02	20.61 ± 10.32	1.98 × 10^−108^
Creatinine	Serum creatinine in mg/dL	1.26 ± 0.52	1.72 ± 1.58	1.28 ± 0.95	3.55 × 10^−46^
Hb	Hemoglobin in g/dL	11.44 ± 1.49	11.19 ± 2.11	11.55 ± 1.27	9.72 × 10^−11^
Platelet	Platelet count in platelets/uL	232,564.16 ± 56,834.95	240,627.10 ± 66,390.62	244,594.27 ± 52,312.73	3.50 × 10^−7^
WBC	White blood cell count in cells/uL	7591.22 ± 1591.14	9718.22 ± 3056.44	8036.06 ± 1626.68	9.85 × 10^−217^
Lymphocyte	Lymphocyte in %	22.08 ± 6.56	16.60 ± 8.54	18.98 ± 7.38	7.23 × 10^−129^
Neutrophil	Neutrophil in %	65.72 ± 8.19	78.07 ± 14.59	73.40 ± 11.29	6.52 × 10^−235^
K	Potassium in mEq/L	3.97 ± 0.32	3.92 ± 0.59	4.06 ± 0.50	2.68 × 10^−17^
Na	Sodium in mEq/L	137.75 ± 1.96	137.71 ± 3.22	138.25 ± 2.74	1.05 × 10^−6^
Smoker (*n*, %)	Daily or occasional smoker	14 (0.42)	150 (1.93)	16 (0.86)	1.40 × 10^−1^
Drinker (*n*, %)	Daily or occasional drinker	23 (0.68)	139 (1.79)	8 (0.43)	6.83 × 10^−4^

**Table 2:** Medical domain-guided augmented dataset characteristics.

Derived Feature	Description	Dementia	Heart Failure	Aortic Valve Disorder	*p*-Value
Body mass index (BMI)	Body fat measurement based on weight in kg divided by the square of height in m	23.37 ± 1.78	24.17 ± 3.37	22.65 ± 2.39	1.13 × 10^−104^
Mid blood pressure	The mean of SBP and DBP	98.36 ± 6.56	97.89 ± 9.03	98.61 ± 6.61	3.43 × 10^−4^
Mean arterial pressure	The average blood pressure over a complete cardiac cycle calculated by adding DBP to one-third of the difference between SBP and DBP	88.45 ± 5.98	87.98 ± 8.37	88.36 ± 6.05	4.74 × 10^−3^
BUN-to-creatinine ratio	The ratio of blood urea nitrogen-to-creatinine	18.36 ± 5.57	20.39 ± 12.94	16.04 ± 6.52	5.44 × 10^−61^
Plasma osmolality	The body’s electrolyte–water balance measurement calculated by (2Na) + (FBS/18) + (BUN/2.8)	291.04 ± 3.36	292.66 ± 8.16	290.09 ± 4.79	4.39 × 10^−62^
Na-to-K ratio	The ratio of sodium and potassium	34.94 ± 2.53	35.85 ± 5.38	34.65 ± 3.68	1.72 × 10^−34^
LDL-to-HDL ratio	The ratio of LDL cholesterol-to-HDL cholesterol	2.51 ± 0.36	2.7 ± 0.49	2.52 ± 0.41	4.54 × 10^−111^
Cholesterol-to-HDL ratio	The ratio of total cholesterol-to-HDL cholesterol	3.84 ± 0.54	3.98 ± 0.64	3.79 ± 0.5	9.20 × 10^−51^
Triglyceride-to-HDL ratio	The ratio of triglyceride level to HDL cholesterol	2.57 ± 0.71	2.82 ± 0.81	2.62 ± 0.8	2.22 × 10^−59^
Non-HDL ratio	Total cholesterol minus HDL cholesterol	129.77 ± 22.48	123.89 ± 18.45	121.36 ± 17.3	8.71 × 10^−64^
Neutrophil count	The estimated neutrophil count calculated using the rule of three applied to the total white blood cell count	5967.54 ± 1297.72	7620.04 ± 2941.34	6196.43 ± 1548.1	2.32 × 10^−271^
Lymphocyte count	The estimated lymphocyte count calculated using the rule of three applied to the total white blood cell count	1633.2 ± 347.41	1507.72 ± 548.78	1514.33 ± 448.91	2.04 × 10^−35^
Neutrophil-to-lymphocyte ratio	The ratio of the estimated neutrophil count to the estimated lymphocyte count	3.78 ± 1.12	5.71 ± 3.25	4.4 ± 1.61	1.16 × 10^−282^
MDRD	The estimated glomerular filtration rate using the MDRD formula	47.07 ± 18.28	47.08 ± 22.35	49.07 ± 20.42	7.94 × 10^−4^
CKD-EPI	The estimated glomerular filtration rate using the CKD-EPI formula	46.63 ± 17.79	46.97 ± 21.9	49.11 ± 19.8	5.83 × 10^−5^
CVD risk score	The estimated 10-year risk score for atherosclerotic CVD	2.33 ± 0.77	2.21 ± 0.79	2.09 ± 0.69	4.52 × 10^−26^

**Table 3:** Descriptive summary of model performance of stratified 10-fold cross-validation.

Model	Baseline Feature Set (*n* = 22)						Feature Set (*n* = 108)					
	Precision (%)	Recall (%)	F1 Score (%)	Accuracy (%)	AUC	AUPRC	Precision (%)	Recall (%)	F1 Score (%)	Accuracy (%)	AUC	AUPRC
XGBoost	90.28 ± 0.91	87.53 ± 1.20	88.79 ± 0.99	91.75 ± 0.64	0.9730 ± 0.0042	0.9452 ± 0.0050	91.31 ± 0.84	88.68 ± 1.15	89.90 ± 0.92	92.56 ± 0.67	0.9806 ± 0.0030	0.9552 ± 0.0050
GB	88.00 ± 1.11	86.82 ± 1.01	87.37 ± 0.97	90.64 ± 0.68	0.9665 ± 0.0040	0.9336 ± 0.0061	89.59 ± 0.83	88.26 ± 1.17	88.87 ± 0.79	91.72 ± 0.49	0.9762 ± 0.0030	0.9469 ± 0.0045
RF	88.52 ± 0.77	87.45 ± 0.82	87.95 ± 0.63	90.95 ± 0.59	0.9708 ± 0.0046	0.9393 ± 0.0065	88.68 ± 0.71	87.53 ± 0.65	88.07 ± 0.50	91.02 ± 0.48	0.9761 ± 0.0029	0.9455 ± 0.0050
ET	86.05 ± 0.90	86.26 ± 0.83	86.12 ± 0.76	89.40 ± 0.64	0.9666 ± 0.0038	0.9325 ± 0.0047	86.51 ± 0.94	86.10 ± 0.51	86.28 ± 0.52	89.63 ± 0.38	0.9703 ± 0.0032	0.9348 ± 0.0046
SVM	78.61 ± 1.30	80.55 ± 1.40	79.43 ± 1.26	83.27 ± 0.94	0.9282 ± 0.0047	0.8674 ± 0.0097	80.01 ± 1.04	81.61 ± 1.31	80.48 ± 0.78	84.64 ± 0.65	0.9407 ± 0.0036	0.8727 ± 0.0093
TabNet	75.36 ± 1.88	79.28 ± 1.47	76.88 ± 1.76	81.00 ± 1.84	0.9173 ± 0.0061	0.8463 ± 0.0136	81.04 ± 1.58	81.40 ± 1.03	81.28 ± 1.25	85.43 ± 1.06	0.9250 ± 0.0055	0.8558 ± 0.0109
DT	80.06 ± 1.24	83.06 ± 1.08	81.33 ± 1.03	85.14 ± 0.93	0.8753 ± 0.0069	0.7231 ± 0.0123	79.75 ± 1.53	82.44 ± 1.57	80.92 ± 1.50	84.89 ± 1.26	0.8707 ± 0.0110	0.7167 ± 0.0189
KNN	76.82 ± 1.15	82.63 ± 1.11	78.95 ± 1.16	82.17 ± 1.05	0.9105 ± 0.0065	0.7961 ± 0.0088	77.37 ± 1.36	83.22 ± 1.30	79.49 ± 1.35	82.70 ± 1.21	0.9178 ± 0.0078	0.8090 ± 0.0154

**Table 4:** Model performance based on test set.

Model	Baseline Feature Set (*n* = 22)						Full Feature Set (*n* = 108)					
	Precision (%)	Recall (%)	F1 Score (%)	Accuracy (%)	AUC	AUPRC	Precision (%)	Recall (%)	F1 Score (%)	Accuracy (%)	AUC	AUPRC
XGBoost	90.20	87.88	88.97	91.76	0.9754	0.9481	91.42	89.08	90.19	92.73	0.9816	0.9570
GB	87.70	87.46	87.57	90.72	0.9695	0.9387	89.34	88.23	88.77	91.69	0.9765	0.9480
RF	88.11	87.68	87.88	90.69	0.9719	0.9423	88.52	88.12	88.32	90.99	0.9747	0.9448
ET	85.64	86.72	86.12	89.15	0.9684	0.9369	86.48	85.76	86.11	89.22	0.9689	0.9341
SVM	79.31	81.97	80.47	83.95	0.9377	0.8762	79.83	82.84	81.10	84.72	0.9435	0.8771
TabNet	76.42	80.42	78.05	81.79	0.9142	0.8469	78.93	80.66	79.72	83.83	0.9255	0.8554
DT	77.66	81.73	79.28	82.99	0.8633	0.6979	78.60	82.69	80.32	83.91	0.8709	0.7099
KNN	74.38	81.26	76.60	79.75	0.9074	0.7862	76.60	82.97	78.75	81.60	0.9132	0.7929

## Data Availability

The datasets presented in this article are not readily available because the data are part of an ongoing study.
